# Utility of Intravascular Ultrasound During Carotid Angioplasty and Stenting with Proximal Protection

**DOI:** 10.7759/cureus.4935

**Published:** 2019-06-18

**Authors:** Simon Morr, Kunal Vakharia, Andrew A Fanous, Muhammad Waqas, Adnan H Siddiqui

**Affiliations:** 1 Neurosurgery, New York-Presbyterian Hospital-Columbia and Cornell, New York, USA; 2 Neurosurgery, Gates Vascular Institute/Kaleida Health, Buffalo, USA; 3 Neurosciences, INOVA Medical Group Neurosurgery, Alexandria, USA; 4 Neurosurgery, Jacobs School of Medicine and Biomedical Sciences, Buffalo, USA

**Keywords:** carotid artery stenting, flow reversal, intravascular ultrasound, proximal embolic protection, thrombus

## Abstract

Carotid artery stenting (CAS) is an established treatment for patients at high-risk for endarterectomy. Patients who undergo CAS have been shown to have periprocedural microembolic events on transcranial Doppler ultrasonography. Flow reversal is often applied in these situations to prevent distal emboli and concurrently allow blood to flush into the common carotid artery. Patients who demonstrate soft plaque morphology that may embolize distally during CAS benefit from flow reversal. Even so, the all-stroke risk in these patients is nearly 1.4%. High-risk patients typically have more difficult plaque morphology; flow reversal decreases the rate of distal emboli but does not offer the intraprocedural visualization seen with intravascular ultrasound (IVUS). In this paper, we illustrate potential periprocedural outcomes associated with stenting of the stenotic carotid bifurcation under flow reversal and how IVUS influenced endovascular management. Three high-risk patients who underwent CAS with direct common carotid artery cutdown approaches due to common carotid ostia disease with flow-reversal proximal embolic protection also had intraprocedural IVUS performed to evaluate plaque morphology and stability before the protection system was removed. Case 1 illustrates no intraluminal thrombus on IVUS, requiring no further intervention after stent placement. Case 2 demonstrates intraluminal thrombus on IVUS requiring a second stent to stabilize plaque. Case 3 shows the inadequate resolution of thrombus after a second stent, which was addressed with balloon angioplasty. In our experience, using IVUS as an adjunct to CAS under proximal embolic protection helped demonstrate plaque morphology and plaque fragmentation after stent placement. These cases illustrate the potential benefit of allowing stabilization of the plaque before flow reversal is stopped.

## Introduction

Carotid artery stenting (CAS) was initially introduced as a treatment alternative for patients deemed high risk for carotid endarterectomy. High-risk factors included medical comorbidities, poor surgical anatomy (such as a high carotid bifurcation, previous neck surgery, spinal immobility, recurrent stenosis), and advanced age [1-3]. Several trials have compared stenting to endarterectomy. The Carotid Revascularization Endarterectomy versus Stenting Trial (CREST) was the largest randomized controlled trial and demonstrated no significant difference in the composite outcome of stroke, myocardial infarction, and death within 30 days [1,4]. The incidence of periprocedural stroke was greater in the stenting group, whereas the incidence of myocardial infarction was greater in the carotid endarterectomy group. Periprocedural microembolic events have been documented with transcranial Doppler ultrasonography in cases of symptomatic atherosclerotic plaque [3-4]. Technological advancements in embolization prevention during CAS are aimed at diminishing adverse periprocedural neurological complications.

Proximal and distal protection systems comprise the two main categories of embolic prevention. Distal protection provides a filtration mechanism for periprocedural embolic material. However, the filter must first be advanced across the lesion, thus permitting distal embolization to occur before it is deployed [5-6]. Proximal protection is intended to establish flow reversal by occluding flow proximal to the lesion, thereby obviating the need to cross the lesion while it is unprotected. The aim is to divert embolic debris proximally into the common carotid artery (CCA), rather than distally into the intracranial vasculature. Nevertheless, concerns about flow stasis and insufficient back pressure from the low-resistance intracranial vascular bed have been expressed [7]. Flow reversal may be insufficient to reliably and systematically address all cases of intraluminal thrombus, especially in the setting of plaque and the often aggressive, intimal manipulation during stenting. A final angiogram for post-stenting luminal visualization may easily dislodge such thrombus intracranially, if not detected earlier.

Intravascular ultrasound (IVUS) is a catheter-based endoluminal imaging technology that in experienced hands can be used to assess intimal plaque and thrombus. Though IVUS is not currently equipped to provide immediate quantitative data, the qualitative evaluation of the visualized plaque architecture is predictive of embolic potential [8-11]. An ex-vivo study has demonstrated good concordance between internal carotid artery (ICA) plaque morphology on IVUS and its histological correlates [12].

IVUS may be superior to even angiography for visualization of multiple small intraluminal plaque herniations between stent tines in patients who have undergone carotid stenting and angioplasty [13-14]. IVUS allows for the opportunity to address this potential embolic source prior to the removal of protection devices and the antegrade high-pressure injection of contrast material for postprocedure/control imaging. This is particularly applicable in cases of proximal protection: while the retrograde flow is maintained, IVUS can be used to assess for both intraluminal thrombus and optimal stent placement, thus permitting the opportunity to perform further interventions and minimize the risk of distal embolic events. We here report three paradigmatic cases of CAS performed under proximal protection that demonstrate the utility and application of this imaging modality to the endovascular armamentarium.

## Materials and methods

The local institutional review board approved the protocol for this retrospective study and the study was performed in accordance with the ethical standards laid down in the 1964 Declaration of Helsinki and its later amendments. The use of the IVUS catheter in this report represents an off-label indication.

Description of IVUS catheter

The IVUS catheter used at our center (Eagle Eye Platinum RX Digital IVUS Catheter, Volcano Corporation, San Diego, CA) has a tapered distal tip that emerges from the hydrophilic-coated lumen ( images found at http://www.volcanocorp.com/products/pdf-files/EEP_data_sheet_rev4.pdf; http://www.volcanocorp.com/products/ivus-imaging/eagle-eye-platinum.php). The IVUS catheter can be advanced via a 5F femoral or brachial access sheath. Radiopaque markers are present at 10-mm intervals, as well as at the probe from which the ultrasonographic image is generated. Although the distal tip is not cannulated, the IVUS allows for passage over a maximum 0.014-in guidewire. Under direct fluoroscopic guidance, the IVUS catheter is placed onto the guidewire that has been previously positioned in the artery. A guidewire of 0.014-in or smaller can be used. The catheter is then advanced over the guidewire to the site of the vasculature to be imaged. After the images are obtained, the catheter is removed.

Description of flow-reversal embolic protection system

The patients described in the illustrative cases presented here underwent carotid stent placement with angioplasty using a direct common carotid artery (CCA) cut-down approach with proximal flow reversal embolic protection. Flow reversal was accomplished with the MICHI Neuroprotection System (Silk Road Medical, Sunnyvale, CA). This system utilizes direct carotid access via a small incision made between the clavicular heads of the sternocleidomastoid muscle. The carotid sheath is connected to a flow regulator and filter, which is continuous with a sheath inserted in the femoral vein. In this way, blood flow is temporarily diverted away from the brain during treatment. Debris created during the procedure is directed away from the brain and trapped in the filter and “debris-free” blood is returned into the femoral vein, thereby decreasing blood loss.

## Results

Following cases were included in the study.

Illustrative case 1

A 70-year-old man with a history of hypertension, type 2 diabetes mellitus requiring scheduled and as-needed insulin, asthma, and coronary artery disease status post previous coronary artery stenting for which he was taking aspirin and clopidogrel, presented from an outside hospital for evaluation of left hemiparesis. He reported approximately a 1-week history of left-lower extremity “heaviness” and numbness in addition to a one-day history of left-upper extremity weakness. Upon arrival at our institution, the patient had a National Institutes of Health Stroke Scale (NIHSS) score of 0 and no focal neurologic deficits. A computed tomographic angiogram was completed, showing severe ICA stenosis.

**Figure 1:**
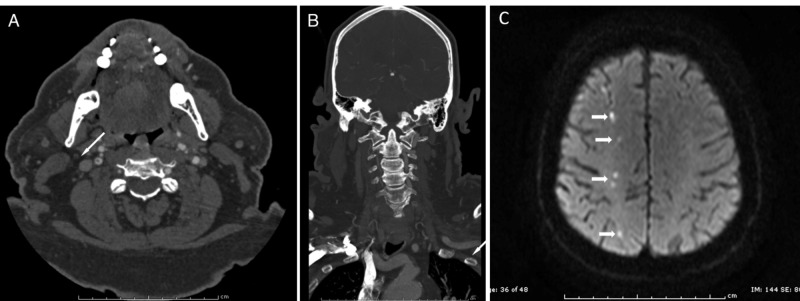
Computed tomographic angiogram and magnetic resonance imaging of Case 1 (A) Computed tomographic angiogram (CTA) of the neck, axial view, demonstrating the “target sign,” indicating intraluminal carotid thrombus and severe carotid artery stenosis (arrow). (B) Neck CTA, coronal view, demonstrating intraluminal brachiocephalic artery thrombus (arrow). (C) Brain magnetic resonance image demonstrating the watershed diffusion restriction pattern (white arrows).

Due to vessel tortuosity in the setting of free thrombus proximal to the lesion as well as high bifurcation (Figure 1A-1C), the patient was taken to the angiography suite for carotid stent placement with angioplasty using a direct carotid cut-down approach given his proximal common carotid artery stenosis with proximal flow reversal embolic protection. As part of the standard protocol at our institution for use of the MICHI Neuroprotection System, the patient was positioned supine with a shoulder roll for mild extension of the cervical spine and enhanced exposure of the neck. The patient was placed under general anesthesia. Once the anatomic landmarks (specifically the sternal notch, clavicle, mastoid, and medial edge of the sternocleidomastoid muscle) were identified, Doppler ultrasound imaging was utilized to locate the common, internal, and external carotid arteries. The distance between the carotid bifurcation and the clavicle was measured. After standard application of betadine and chlorhexidine gluconate and standard surgical draping for carotid endarterectomy, a linear incision was made above the clavicle between the sternal and clavicular heads of the sternocleidomastoid muscle. Dissection with bipolar cautery and Metzenbaum scissors was continued until the CCA was identified and readily palpated. The edges of the carotid sheath were elevated with tack-up sutures.

**Figure 2:**
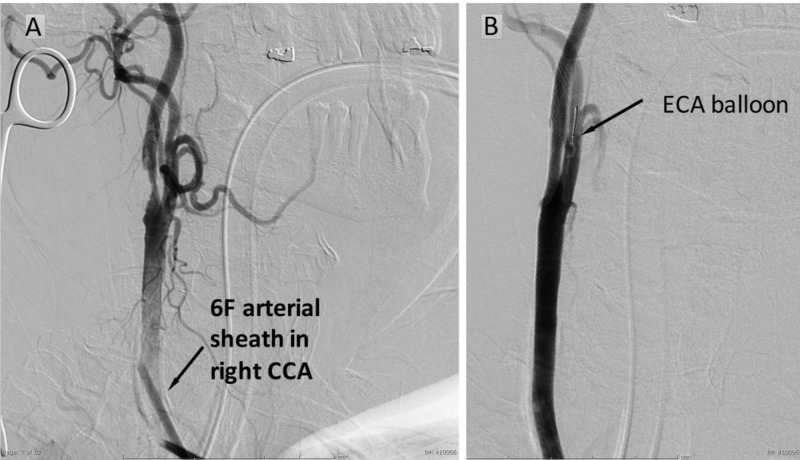
Digital subtraction carotid angiography of Case 1 (A) Diagnostic lateral projection angiogram demonstrating a 6F arterial sheath inserted into the right common carotid artery confirmed severe stenosis at the carotid bifurcation. (B) Lateral projection angiogram demonstrating an external carotid artery (ECA) balloon (Silk Road Medical, Sunnyvale, CA) in the proximal ECA

6-French (F) left femoral venous sheath was placed before direct micropuncture of the exposed CCA and a contrast angiographic run was obtained, demonstrating significant carotid stenosis (Figure 2A). A bolus dose of heparin was administered with the goal of an activated coagulation time (ACT) above 250 seconds. The right CCA was then accessed using a 21-gauge micropuncture needle. This was exchanged for an 8F dilator over a 0.018-inch microwire. Under roadmap guidance, a 0.035-inch wire (Terumo Corporation, Tokyo, Japan) was advanced into the external carotid artery (ECA) and the 8F dilator was replaced with an 8F arterial sheath from the MICHI system. A Rummel tourniquet was then placed around the CCA and a purse-string 5-0 prolene suture was placed in the carotid adventitia. The right CCA was accessed using a 5F micropuncture needle. A dilator sheath was exchanged for the micropuncture needle over a microwire. The 8F arterial sheath was exchanged for a dilator sheath over a J-wire. Under roadmap guidance, a Gore external carotid balloon (W.L. Gore and Associates, Flagstaff, AZ) was positioned and inflated in the right ECA concomitantly with the MICHI system (Figure 2B). The Rummel tourniquet was tightened, and flow reversal was established by connecting the arterial and venous sheaths with the interceding embolization filtration system.

**Figure 3:**
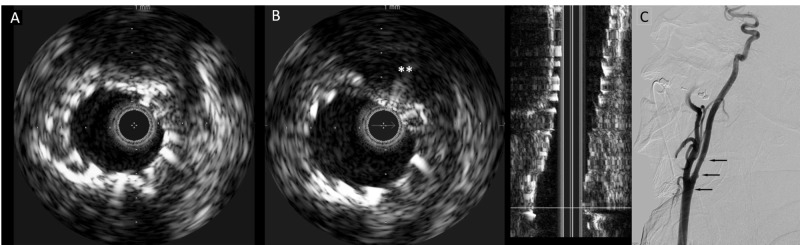
Intravascular ultrasound and digital subtraction carotid angiogram of Case 1 (A) Poststenting intravascular ultrasound image demonstrating good apposition of the stent to the vessel wall. (B) Thrombus is shown to be trapped (asterisks) by the deployed stent, without evidence of debris inside the stent. (C) Final lateral projection angiogram demonstrating significantly improved vessel caliber (arrows)

The stenotic lesion was crossed with a Spartacore 300 microwire (Abbott Vascular Inc., Santa Clara, CA), and an 8-mm × 36-mm Wallstent (Boston Scientific, Natick, MA) was placed across the luminal stenosis. The IVUS catheter was then introduced to examine intraluminal status post-stenting (Figure 3A). No intraluminal thrombus was noted inside the stent through the radiopaque probe of the IVUS catheter (Figure 3B). Poststenting angiography demonstrated marked diminution of the stenosis (Figure 3C). The sheath was then removed, and the purse-string suture was tied down. The patient remained neurologically stable throughout the procedure as monitored by electroencephalography (EEG).

Illustrative case 2

**Figure 4:**
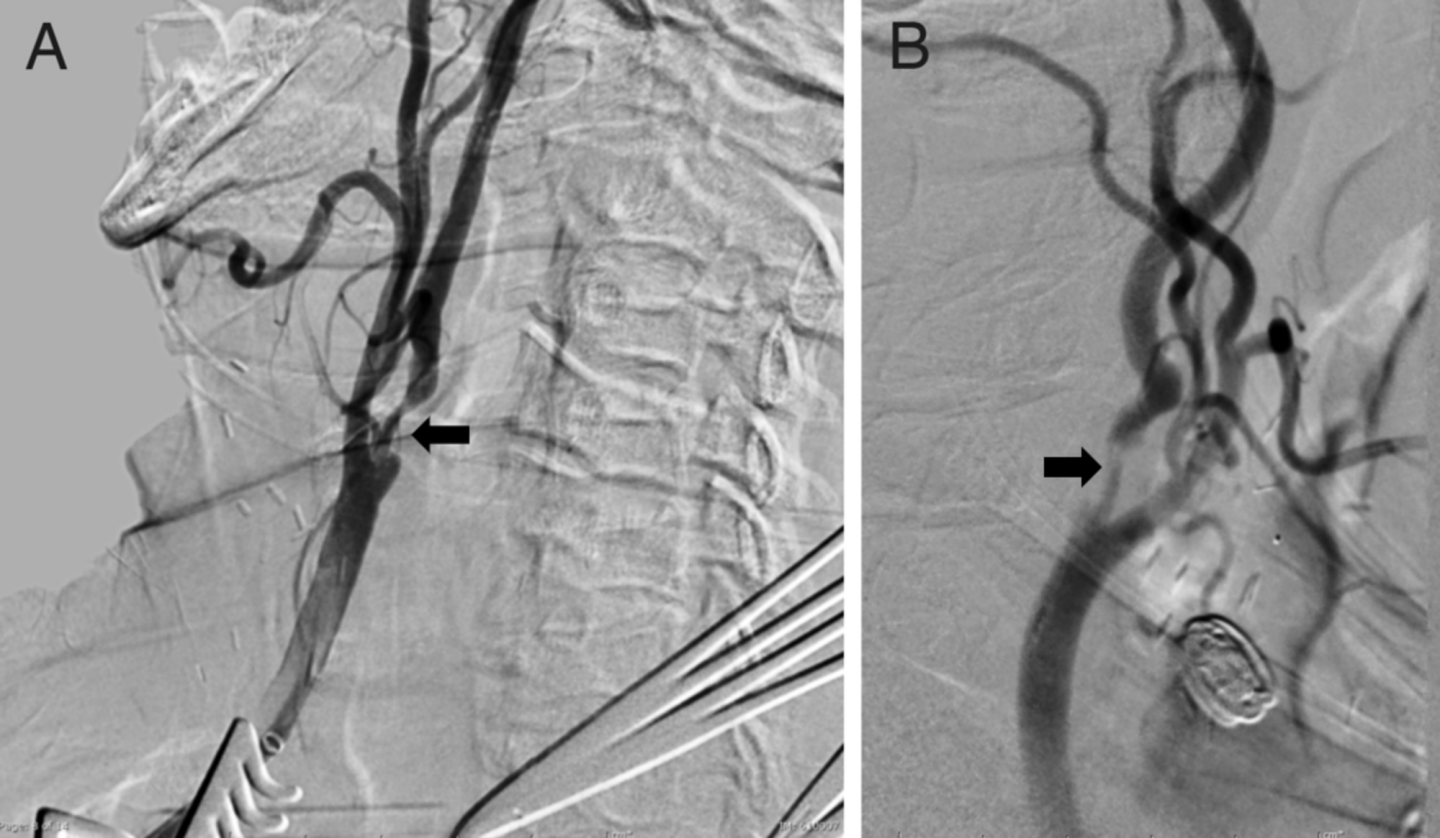
Diagnostic lateral and oblique projection angiographic images of Case 2 Diagnostic lateral (A) and oblique (B) projection angiographic images confirming severe stenosis at the carotid bifurcation.

A 60-year-old man with a previous history of bilateral carotid endarterectomies and recent leg claudication was found to have >75% stenosis, recurrent right ICA stenosis on an aortic arch run on a recent angiogram (Figures 4A-4B) performed for evaluation and subsequent stenting of the external iliac arteries. His history was also remarkable for cardiac stenting 4 years back, carotid endarterectomy 8 years back, and coronary artery bypass grafting 14 years back. Medications included aspirin, clopidogrel, and rosuvastatin. The patient was referred to the neurosurgery service for evaluation of carotid artery restenosis.

**Figure 5:**
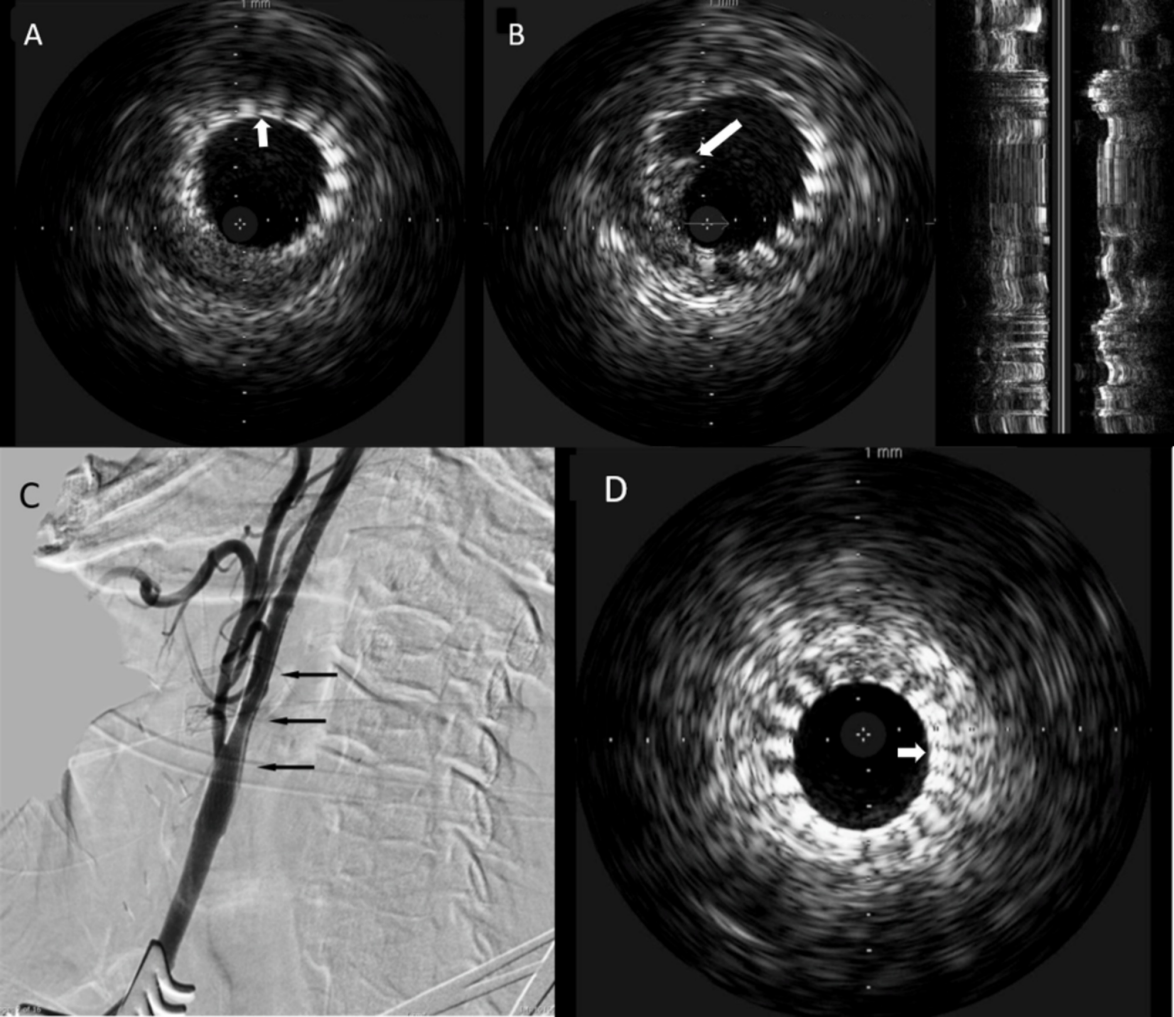
Poststenting intravascular ultrasound and digital subtraction carotid angiogram of Case 2 (A) Poststenting intravascular ultrasound image demonstrating good wall apposition of the stent (arrow). (B) Intraluminal debris noted inside stent (arrow). (C) Resolution of thrombus noted after placement of the second stent (arrow). (D) Final lateral projection diagnostic angiogram demonstrating significantly improved vessel caliber (arrows).

Given the anticipated difficulty associated with femoral artery access because of the presence of severe bilateral iliac artery stenotic disease, carotid access was obtained using the CCA cut-down technique as described above in case 1. The right CCA was accessed with a 4F micropuncture needle, and the left femoral vein was accessed with an 8F sheath. After an initial diagnostic angiogram was performed that demonstrated the same degree of stenosis and high bifurcation as well as prior carotid endarterectomy site, a Spartacore 300 microwire was advanced into the distal right cervical ICA. An 8-mm × 6-mm ×40-mm Xact stent (Abbott Vascular Inc.) was advanced over the microwire and deployed in the stenotic lesion (Figure 5A). The IVUS catheter was then introduced and advanced and intraluminal debris was noted on the images (Figure 5B). At that time, a 6-mm × 8-mm × 30-mm Xact stent was advanced and deployed over the previously placed stent with minimal overlap (Figure 5C). The IVUS catheter was again advanced over the microwire and the IVUS images revealed the resolution of the debris. A poststent angioplasty was performed with a 4-mm × 30-mm Aviator balloon (Cordis, Bridgewater Township, NJ). Postprocedure angiography demonstrated considerable diminution of stenosis (Figure 5D). The patient remained neurologically stable throughout the procedure as monitored by EEG.

Illustrative case 3

A 79-year-old man was referred to the neurosurgery service by his ophthalmologist to whom the patient presented for visual difficulties that were likely diabetes-related. The patient reported a prolonged and progressive history of multiple syncopal events per week and episodic transient left-sided weakness. Previous medical history included the placement of five coronary artery stents, type 2 diabetes mellitus for which he was placed on scheduled insulin, hypertension, and chronic kidney disease. Relevant medications at the time of presentation included aspirin, clopidogrel, and rosuvastatin.

**Figure 6:**
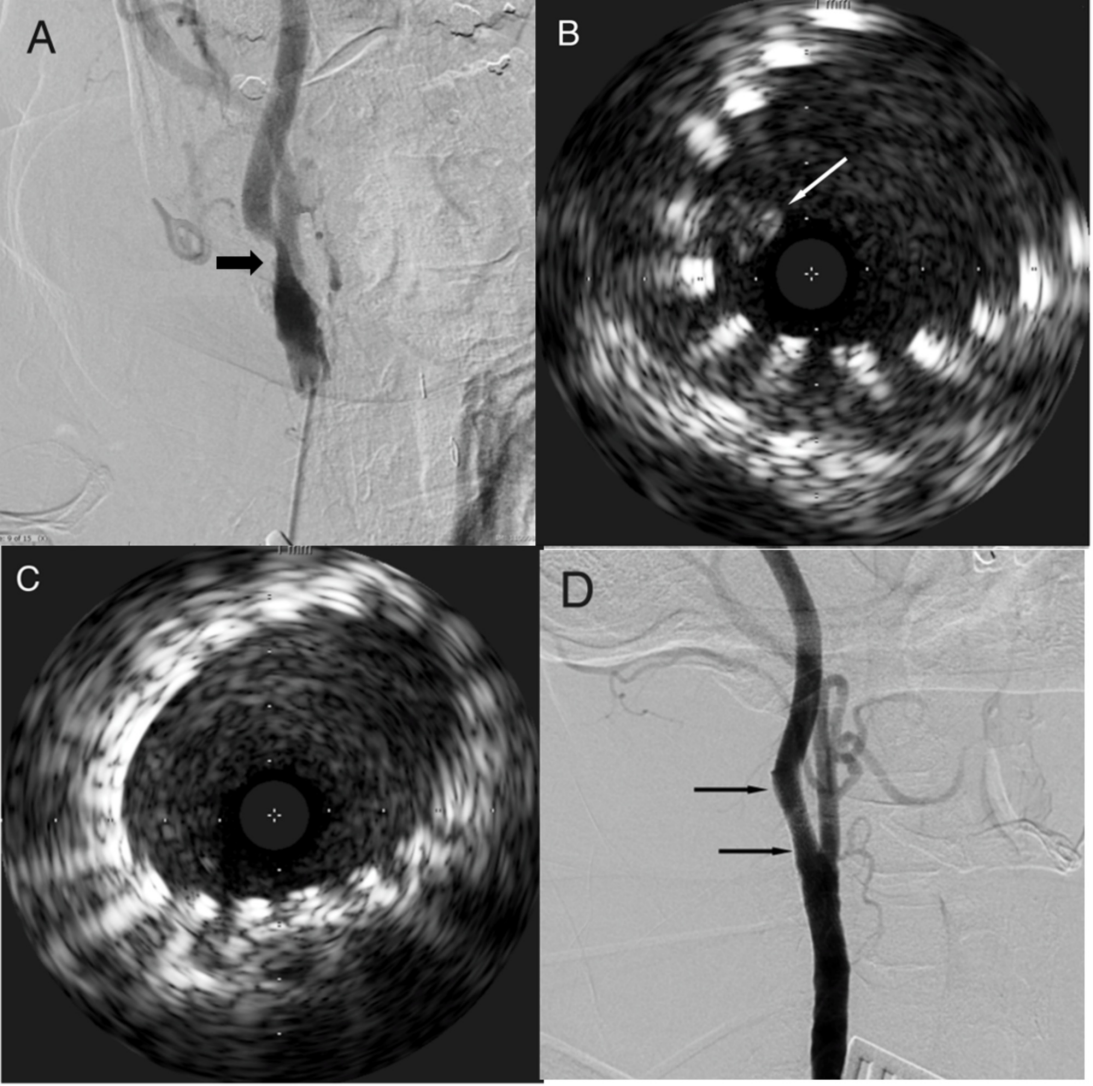
Digital subtraction carotid angiogram and intravascular ultrasound (IVUS) of Case 3 (A) Lateral projection diagnostic angiogram confirming severe stenosis (arrow) in the internal carotid artery just distal to the bifurcation. (B) Poststenting intravascular ultrasound (IVUS) image demonstrating thrombus inside the stent (arrow). (C) Final IVUS image after second stent deployment and balloon angioplasty demonstrating resolution of thrombus. (D) Final lateral projection angiogram demonstrating improved vessel caliber (arrows).

Carotid duplex imaging revealed an 80%-99% stenosis and a peak velocity of 263 cm per second on the right and 60%-80% stenosis and a peak velocity of 151 cm per second on the left. Brain magnetic resonance imaging (MRI) demonstrated no evidence of acute infarct. A diagnostic angiogram demonstrated severe (81%) right ICA stenosis (Figure 6A). Owing to the substantial vessel tortuosity and high bifurcation documented by angiography, endovascular access was obtained using the CCA cut-down technique as described above in case 1. The right CCA was accessed with a 4F micropuncture needle and the left common femoral vein was accessed with a 5F sheath. The stenotic ICA lesion was crossed with a Spartacore 0.14-inch micro guidewire (Abbott Vascular Inc.). A 6-mm × 8-mm × 30-mm Xact stent was deployed. An IVUS examination demonstrated intraluminal thrombus (Figure 6B), and another 6-mm × 8-mm × 30-mm Xact stent was deployed over the first with considerable ease. Repeat IVUS examination demonstrated a small fragment of thrombus that persisted despite multiple aspiration attempts in the setting of flow reversal. A 4-mm × 20-mm Aviator balloon was advanced through the plaque and angioplasty was performed. Repeat IVUS examination demonstrated complete resolution of the thrombus (Figure 6C). Repeat angiography demonstrated a patent ICA lumen and appropriately positioned stents (Figure 6D). The patient remained neurologically stable throughout the procedure as monitored by EEG.

## Discussion

In this report, we described three scenarios that illustrate potential periprocedural outcomes associated with stenting of the stenotic carotid bifurcation under flow reversal. These three outcomes would not be recognized prior to the cessation of flow reversal without the use of IVUS. Post-stenting angiography for evaluation of these lesions may have been too late as the thrombus may have been pushed forward into the intracranial vasculature. Case 1 illustrates no intraluminal thrombus on IVUS, requiring no further intervention. Case 2 illustrates intraluminal thrombus that was adequately addressed with a second stent. Case 3 illustrates an inadequate resolution of thrombus after a second stent was placed, and this condition was addressed with balloon angioplasty.

Although flow reversal avoids the potential for embolization associated with distal protection at the time of initially crossing the lesion, it most aptly protects against embolization as long as the blood remains in a state of retrograde flow. Poststenting antegrade angiography to characterize the poststenting intraluminal anatomy and stent position may itself dislodge and/or carry embolic material distally, which may contribute to increasing the stroke risk associated with endovascular carotid interventions. IVUS in conjunction with proximal protection minimizes the risk of distal embolization of intraluminal thrombus after stenting by identifying the thrombus prior to the cessation of flow reversal and antegrade injection of contrast material.

Systematic evaluation of the utility of IVUS in trials with more patients remains necessary to evaluate for clinical relevance. Correlation with previous embolic events noted on transcranial Doppler and magnetic resonance imaging would be helpful in more clearly delineating the source of periprocedural stroke despite proximal protection. Prevalence of the respective three scenarios here presented will be useful to delineate the cost-effectiveness of IVUS. Further multicenter trials should also address and account for the learning curve associated with the technology and the required experience to successfully implement it with benefit. We believe technologies aimed at diminishing periprocedural embolic events hold great potential for diminishing stroke risk associated with CAS. Here, we have demonstrated the clinical utility of IVUS as an adjunct to CAS performed under proximal embolization protection.

## Conclusions

We believe technologies aimed at diminishing periprocedural embolic events hold great potential for diminishing stroke risk associated with CAS. Here, we have demonstrated the clinical utility of IVUS as an adjunct to CAS performed under proximal embolization protection.
